# Colonic Delivery of Nutrients for Sustained and Prolonged Release of Gut Peptides: A Novel Strategy for Appetite Management

**DOI:** 10.1002/mnfr.202200192

**Published:** 2022-08-19

**Authors:** Remi Kamakura, Ghulam Shere Raza, Nalini Sodum, Vesa‐Pekka Lehto, Miia Kovalainen, Karl‐Heinz Herzig

**Affiliations:** ^1^ Research Unit of Biomedicine Faculty of Medicine, and Medical Research Center University of Oulu and Oulu University Hospital Oulu 90220 Finland; ^2^ Department of Applied Physics Faculty of Science and Forestry University of Eastern Finland Kuopio FI‐70211 Finland; ^3^ Department of Pediatric Gastroenterology and Metabolic Diseases Pediatric Institute Poznan University of Medical Sciences Poznań 60–572 Poland

**Keywords:** appetite, delivery systems, GLP‐1, nutrients, obesity

## Abstract

Obesity is one of the major global threats to human health and risk factors for cardiometabolic diseases and certain cancers. Glucagon‐like peptide‐1 (GLP‐1) plays a major role in appetite and glucose homeostasis and recently the USFDA approved GLP‐1 agonists for the treatment of obesity and type 2 diabetes. GLP‐1 is secreted from enteroendocrine L‐cells in the distal part of the gastrointestinal (GI) tract in response to nutrient ingestion. Endogenously released GLP‐1 has a very short half‐life of <2 min and most of the nutrients are absorbed before reaching the distal GI tract and colon, which hinders the use of nutritional compounds for appetite regulation. The review article focuses on nutrients that endogenously stimulate GLP‐1 and peptide YY (PYY) secretion via their receptors in order to decrease appetite as preventive action. In addition, various delivery technologies such as pH‐sensitive, mucoadhesive, time‐dependent, and enzyme‐sensitive systems for colonic targeting of nutrients delivery are described. Sustained colonic delivery of nutritional compounds could be one of the most promising approaches to prevent obesity and associated metabolic diseases by, e.g., sustained GLP‐1 release.

## Graphical abstract

Gut peptides GLP‐1 and PYY are secreted from the gastrointestinal tract after a meal and play a major role in appetite regulation. These peptides are degraded immediately after secretions. Colonic delivery of nutrients will increase GLP‐1 and PYY secretion in a sustained manner. This would be a novel approach to reduce appetite and prevent/treat obesity.

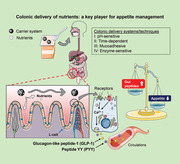

## 1 Obesity and Appetite Regulation

Obesity is one of the major global threats to human health. Its prevalence has almost tripled since 1975.^[^ ^1^ ^]^ In 2015, 4 million deaths have been reported globally related to high body mass index (BMI: kg m^−2^).^[^ ^2^ ^]^ A clear positive correlation between BMI of over 30 and the mortality risk has been reported.^[^ ^3^ ^]^ Various strategies such as diet, physical activity, pharmacotherapy, and bariatric surgery are used for losing weight. Increased physical exercise and changes in the nutritional load are considered as key factors for reducing body weight. However, exercise is often too difficult and demanding for subjects at risk and thus seldom sufficient to change the risk profile. Hence, multimodal therapy is necessary consisting of exercise, nutrition, and appetite suppressing medications.

Appetite is regulated by neural and humoral signals via gut‐brain communications. The arcuate nucleus (ARC) of the hypothalamus and brainstem are the central regulators for energy homeostasis.^[^ ^4^ ^]^ Signals from the periphery stimulate the neuronal subpopulations of ARC, regulating energy homeostasis and food intake.

Gastrointestinal (GI) hormones such as glucagon‐like peptide‐1 (GLP‐1), peptide YY (PYY), cholecystokinin (CCK), and ghrelin influence pre‐ and postprandial appetite regulation.^[^ ^5^ ^]^ Ghrelin is secreted from X/A‐like cells of the gastric fundus and stimulates appetite. The plasma concentrations of ghrelin are highest in the fasting period.^[^ ^6^ ^]^ CCK is secreted from the I‐cells in response to intraluminal nutrients, delays gastric emptying, enhances the release of pancreatic enzymes, and stimulates gallbladder contraction.^[^ ^4^, ^7^ ^]^ GLP‐1 and PYY are secreted from L‐cells in the distal ileum and colon in response to food ingestion and inhibit food intake.^[^ ^8^, ^9^ ^]^ Both GLP‐1 and PYY are well‐known mediators of the ileal brake; the feedback mechanism inhibiting the GI motility, which plays an important role in eating behavior and satiety.^[^ ^10^, ^11^ ^]^ Various studies in animals and humans demonstrated that GLP‐1 has multiple effects such as suppressing food intake, delaying gastric emptying, and stimulating insulin secretion from pancreatic β‐cells.^[^ ^12^ ^]^ The digested nutrients directly interact with their receptors on L‐cells of the GI tract and stimulate GLP‐1 and PYY secretion.^[^ ^13^ ^]^ Though, the majority of digested nutrients are absorbed in the upper GI tract and do not reach the distal ileum and colon where L‐cells are highly abundant.^[^ ^9^ ^]^ Hence, delivering nutrients to the distal GI tract may prolong endogenous gut peptides release, and suppress food intake which might lead to a reduction of body weight. GLP‐1 receptor agonists on the market are for the treatment of obesity and type 2 diabetes.^[^ ^14^ ^]^ However, these drugs are only available for patients with the diagnosis of obesity (ICD‐10 E66) or type 2 diabetes (ICD‐10 E11), and their cost and side effects are currently limiting their broad use.^[^ ^15^ ^]^ Hence, utilizing nutritional compounds to stimulate the release of peptides from the enteroendocrine system to diminish food intake would be a novel approach to manage appetite, and prevent overweight/obesity. The review article focuses on nutrients that endogenously stimulate GLP‐1 and PYY secretion via their receptors in order to decrease appetite as preventive action. In addition, various delivery technologies such as pH‐sensitive, mucoadhesive, time‐dependent, and enzyme‐sensitive systems for colonic delivery of nutrients are described.

## 2 Endocrine Mediators for Appetite Management

### 2.1 Enteroendocrine Cells (EECs)

**Figure 1:**
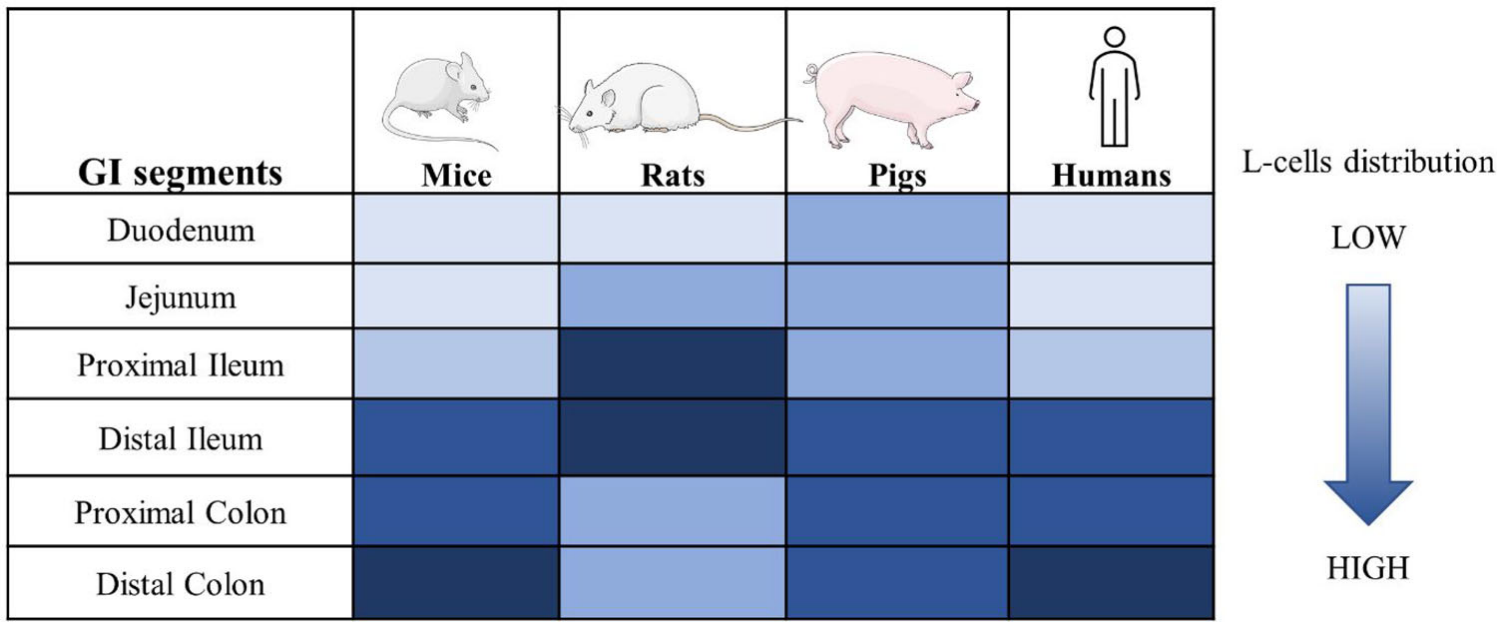
L‐cells distribution in the intestinal tract of mouse, rat, pig, and human.^[^ ^24^, ^25^, ^26^ ^]^

Enteroendocrine cells (EECs) secrete about 30 different peptides into the bloodstream and account for less than 1% population of the total epithelial cells.^[^ ^16^, ^17^, ^18^, ^19^ ^]^ Even though their population is relatively small, they play significant physiological roles in glucose homeostasis, appetite, adiposity, gut motility, and epithelial cells proliferation.^[^ ^20^ ^]^ EECs were initially classified according to their secreting hormones, such as secretin secreting S‐cells, GLP‐1 secreting L‐cells, and glucose‐dependent insulinotropic polypeptide (GIP) secreting K‐cells.^[^ ^21^ ^]^ However, recent studies demonstrated that EECs co‐express and secrete several combinations of gut peptides.^[^ ^22^ ^]^ For example, L‐cells in the upper small intestine co‐secrete GLP‐1 and GIP, whereas in the distal ileum and colon mainly secrete GLP‐1, PYY, and CCK.^[^ ^9^, ^23^ ^]^ Interestingly, L‐cells distribution is distinct among species as shown in **Figure** **1**. In humans and mice, L‐cells are localized in the distal part of the GI tract, in pigs they extend from the duodenum to colon with a higher expression in the distal ileum and colon, whereas in rats they are mainly located in the jejunum and ileum.^[^ ^24^, ^25^, ^26^ ^]^ Hence, the targeted site of action varies between species to stimulate the secretion of gut peptides. GLP‐1 and PYY are co‐secreted from L‐cells, however, the therapeutic effects of PYY on appetite management are still not fully established. Therefore, this review is mainly focused on the GLP‐1 as an appetite‐regulating peptide.

### 2.2 Role of GLP‐1 and PYY in Appetite Regulation

GLP‐1 and PYY are of specific interest for obesity and diabetes management due to their implication in food intake and insulin secretion.^[^ ^27^ ^]^ GLP‐1 is a post‐translational product of proglucagon, secreted from enteroendocrine L‐cells of the intestine in response to nutritional, hormonal, and neuronal stimuli.^[^ ^28^ ^]^ Proglucagon is tissue‐specifically cleaved into several fragments by enzymes, and GLP‐1 is formed in the intestine and brain by prohormone convertase 1/3.^[^ ^29^ ^]^ GLP‐1 secretion in response to nutrients ingestion is biphasic: the initial GLP‐1 release occurs within 15 min by a neural reflex, nutrients, and/or other circulating hormones, while the second additional peak is observed when digested nutrients stimulate the L‐cells in the distal small intestine.^[^ ^30^, ^31^, ^32^ ^]^ The anorexigenic effects of GLP‐1 are mediated via vagal nerves; after truncal vagotomy, these effects are no longer observed in humans.^[^ ^33^ ^]^ Anorectic effects and body weight lowering effects of GLP‐1 are mediated via a central mechanism.^[^ ^34^, ^35^ ^]^ GLP‐1 exerts its anorectic effects by binding to GLP‐1 receptor (GLP‐1R) in the brainstem,^[^ ^36^ ^]^ and studies in mice lacking neuronal GLP‐1R did not produce anorexia after GLP‐1R agonists treatment.^[^ ^37^ ^]^

PYY belongs to the pancreatic polypeptide (PP) family which also includes the neuropeptide Y (NPY) and PP and is a potent appetite‐regulating hormone.^[^ ^38^ ^]^ The pancreatic islet and gigantocellular reticular nucleus of the rostral medulla also produce PYY but to a lesser amount.^[^ ^39^ ^]^ Plasma levels of PYY increase within 15–30 min after a meal, peak at approximately 60–90 min, and remain elevated for up to 6 h.^[^ ^40^ ^]^ Animal and human studies demonstrated that PYY reduces food intake by increasing satiety and delaying gastric emptying.^[^ ^40^, ^41^ ^]^ PYY_3‐36_ binds selectively to Y2 receptors, whereas PYY_1‐36_ binds to Y1‐Y5 receptors, inhibiting food intake.^[^ ^42^ ^]^ The ameliorating role of PYY in obesity and diabetes made it a target to design more potent analogs selective to Y2 receptors, which are resistant to inactivation by enzyme DPP‐4 cleavage.^[^ ^43^ ^]^ Several strategies, such as N‐methyl amino acid incorporation and selective amino acid combinations, have been identified as a stable peptide, however finding long‐acting analogs are still a challenge.^[^ ^44^ ^]^

### 2.3 Nutrients‐Sensing Receptors in L‐Cells

**Figure 2:**
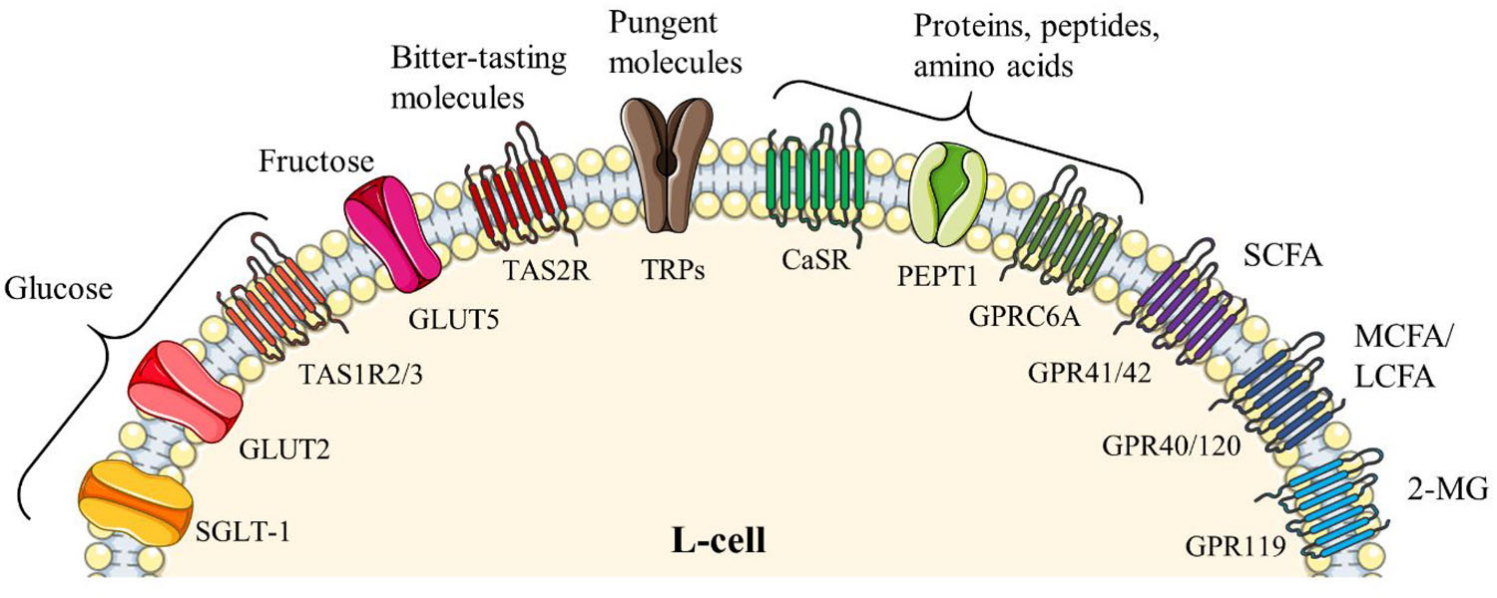
Nutrients and their sensing receptors and transporters on L‐cells.^[^ ^12^, ^177^ ^]^ 2‐MG, 2‐monoacylglycerol; CaSR, calcium‐sensing receptors; FFAR3/FFAR2, GPR40/120, FFAR1/FFAR4; GLUT2, glucose transporter 2; GLUT5, glucose transporter 5; GPRC6A, G protein‐coupled receptor class C group 6 membrane A; GPR41/42, fatty acid receptor 3; LCFA, long‐chain fatty acid; MCFA, medium‐chain fatty acid; PEPT1, peptide transporter‐1; SCFA, short‐chain fatty acid; SGLT‐1, sodium‐coupled glucose cotransporter‐1; TAS1R2/3, sweet taste receptors; TAS2R, bitter taste receptor; and TRPs, transient receptor potential channels.

L‐cells express several transporters and receptors on the cell surface to sense and respond to the luminal nutrients (**Figure** **2**). The digestion products such as glucose, fatty acid, and amino acids act as stimuli for L‐cells via different sensory proteins such as G protein‐coupled receptors (GPRs), ion channels, and transporters. Sodium‐glucose cotransporter‐1 (SGLT1), glucose transporter 2 (GLUT2), and sweet taste receptors (TAS1R2 and TAS1R3) have been identified in the GI tract and mediate glucose‐induced gut peptides secretion.^[^ ^45^, ^46^, ^47^, ^48^, ^49^ ^]^ On the other hand, glucose transporter 5 (GLUT5) stimulates fructose‐induced GLP‐1 secretion.^[^ ^50^ ^]^ Recently, it has been shown that stimulation of the bitter taste receptors (TAS2Rs) modulates EECs secretion and regulates food intake.^[^ ^51^ ^]^ The transient receptor potential (TRP) superfamily are known to be involved in pungent compounds‐induced GLP‐1 secretion in in vitro and animal studies.^[^ ^52^, ^53^ ^]^

Protein and peptides stimulate gut peptide secretion via a calcium‐sensing receptor (CaSR), GPR‐C6A, and peptide transporter‐1 (PEPT1).^[^ ^54^, ^55^ ^]^ Various amino acids including phenylalanine and glutamine stimulated GLP‐1 secretion via CaSR.^[^ ^56^ ^]^ Peptone (protein hydrolysate) has been shown to induce GLP‐1 secretion from L‐cells of mouse primary small intestine by CaSR activation.^[^ ^57^ ^]^ Other amino acids such as arginine, ornithine, and lysine increased GLP‐1 secretion from GLUTag cells via the GPR‐C6A receptor.^[^ ^55^ ^]^ Oligopeptides have been also demonstrated to induce GLP‐1 secretion via PEPT1 in L‐cells isolated from GLU‐Venus transgenic mice.^[^ ^54^ ^]^ In addition, a recent study in human subjects reported that peptides secreted from L‐cells are essential for glucose‐induced GLP‐1 secretion by activation of L‐cells via a paracrine effect.^[^ ^58^ ^]^

L‐cells expressed several GPRs for sensing fatty acids, i.e., GPR41 (fatty acid receptor 3, FFAR3), GPR42 (FFAR2), GPR40 (FFAR1), and GPR120 (FFAR4).^[^ ^59^ ^]^ GPR41 and GPR43 mediate the signals from short‐chain fatty acids (SCFAs), while GPR40 and GPR120 interact with medium‐chain fatty acids (MCFAs), and long‐chain fatty acids (LCFAs).^[^ ^60^ ^]^ Furthermore, GPR119 is involved in GLP‐1 secretion stimulated by LCFA derivatives, and 2‐monoacylglycerol (2‐MG), which is formed during dietary fat digestion.^[^ ^61^, ^62^ ^]^

## 3 Effects of Nutrients and Food Components

Nutrient‐stimulated gut peptide release from enteroendocrine cells is well documented.^[^ ^63^ ^]^ Various studies in animals and humans demonstrated that macronutrients carbohydrates, fats, and proteins all are involved in meal‐stimulated GLP‐1 release.^[^ ^8^, ^64^ ^]^

### 3.1 Saccharides

Saccharides are one of the most known nutritional compounds, stimulating gut hormones release. Especially, glucose is a potent GLP‐1 secretagogue compared to other monosaccharides.^[^ ^8^ ^]^ It has been demonstrated that glucose‐stimulated intestinal GLP‐1 secretion via SGLT1 or GLUT2, and its effect was abolished in SGLT1 or GLUT2 knockout mice.^[^ ^65^, ^66^ ^]^ Fructose increased plasma GLP‐1 concentrations in mice, rats, and healthy human subjects, while in humans its effect was less compared to isocaloric glucose.^[^ ^50^ ^]^ In addition, oral galactose rapidly increased plasma GLP‐1 concentrations in healthy human subjects.^[^ ^67^ ^]^ Disaccharides, i.e., sucrose and isomaltulose also induced GLP‐1 secretion in humans and rodents, respectively.^[^ ^68^, ^69^ ^]^ Saccharides induced GLP‐1 secretion by stimulating the vagal nerve after absorption of saccharides in the upper GI tract.^[^ ^70^ ^]^ Studies in animals and humans demonstrated that SGLT1 inhibition reduces the absorption of intestinal glucose and stimulates the second phase GLP‐1 secretion by other mechanisms, i.e., fermentation products of undigested carbohydrates.^[^ ^71^, ^72^ ^]^

### 3.2 Dietary Fibers (DFs) and SCFAs

Dietary fibers are undigested carbohydrates that offer a natural dietary strategy for reducing calorie intake and inducing satiety.^[^ ^73^ ^]^ Viscous DFs such as β‐glucan, alginate, guar gum, and psyllium increase gut content, slow gastric emptying,^[^ ^74^ ^]^ and stimulate PYY and GLP‐1 secretion.^[^ ^8^ ^]^ Non‐viscous DFs fructo‐oligosaccharide have been shown to reduce energy intake and increase subjective satiety in both humans and rodents.^[^ ^75^, ^76^ ^]^ A meta‐analysis reported that viscous DFs are more potent in reducing food intake compared to non‐viscous DFs.^[^ ^77^ ^]^ Various studies have shown that soluble DFs consumption increases circulating concentrations of the GLP‐1 and PYY in animals and humans.^[^ ^78^, ^79^ ^]^

The bacterial fermentation of DFs in the colon produces SCFAs; acetate, propionate, and butyrate, which stimulate GLP‐1 and PYY secretions via fatty acid receptor GPR43 and GPR41.^[^ ^80^ ^]^ Acetate is the most abundant SCFAs in the circulation taken up by the liver and other tissues for energy sources but also serve as a substrate for the synthesis of cholesterol and LCFAs.^[^ ^81^ ^]^ Small amounts of acetate cross the blood‐brain barrier,^[^ ^82^ ^]^ suggesting that colonic acetate may also reduce appetite directly via anorectic signals in the hypothalamus. Vascular and luminal stimulation with acetate and butyrate increased GLP‐1 and PYY secretion from the isolated perfused rat colon.^[^ ^83^ ^]^ Propionate has been shown to stimulate GLP‐1 and PYY secretion from primary murine colonic cultures and after intra‐colonic administration in rodents.^[^ ^84^ ^]^ Chambers et al.^[^ ^85^ ^]^ reported that targeted delivery of propionate to the colon increased plasma GLP‐1 and PYY levels and decreased food intake in overweight and obese subjects. Furthermore, supplementation of butyrate has been shown to prevent and treat insulin resistance in high‐fat diet‐fed mice.^[^ ^86^ ^]^ Studies in animals demonstrated that butyrate reduces weight gain by inhibiting food intake via gut‐brain neural circuits.^[^ ^87^ ^]^ In addition, several animal studies reported that the anorectic response of butyrate was due to stimulation of GLP‐1 and PYY secretions.^[^ ^80^ ^]^

### 3.3 Lipids

Fats are highly effective in inhibiting appetite and energy intake by stimulating GLP‐1 and GIP secretions.^[^ ^88^ ^]^ Dietary lipids are mainly triglycerides, which are broken down in the intestine into glycerol and free fatty acid. The stimulatory effects of dietary lipids on incretin hormone secretion are dependent on fatty acid chain length and saturation.^[^ ^89^ ^]^ LCFAs are potent GLP‐1 secretagogue by activating the GPR40 and GPR120 receptors.^[^ ^90^, ^91^ ^]^ Administration of sodium oleate (C18:1) into duodenum resulted in a marked GLP‐1 increase in humans, whereas sodium caprylate (C8:0) infusion was ineffective.^[^ ^92^ ^]^ The reduction in energy intake and hunger was more pronounced with an infusion of lauric acid (C12:0) than decanoic acid (C10:0),^[^ ^89^ ^]^ and even reduction in food intake was higher with unsaturated compared to saturated fatty acid in humans.^[^ ^93^ ^]^ Unsaturated fatty acids are more effective in stimulating GLP‐1 secretions than saturated fatty acids in animals and humans.^[^ ^94^, ^95^ ^]^ Especially, long‐chain polyunsaturated fatty acids (LCPUFAs) such as docosahexaenoic acid (DHA, 22:6, n‐3), α‐linolenic acid (αLA, C18:3, n‐3), and eicosapentaenoic acid (EPA, 20:5, n‐3) have been studied. They have been shown to have a higher potency than other LCFA in suppression of body weight gain and appetite via activation of GPR40 and/or GPR120.^[^ ^96^ ^]^ DHA supplementation for 12 weeks reduced fat and carbohydrate intake compared to oleic acid (C18:1, n‐9) in overweight and obese women.^[^ ^97^ ^]^ αLA potently stimulated GLP‐1 secretion and reduced blood glucose in normal and diabetic rats.^[^ ^98^ ^]^ Previous studies reported that targeted delivery of the bioactive compounds to the colon using a limited dose range stimulated GLP‐1 secretions in animals and humans.^[^ ^99^, ^100^, ^101^ ^]^ Adachi et al.^[^ ^98^ ^]^ administered αLA at a dose of 300 nmol 100 µL^−1^ into the different segment of intestine like duodenum, ileum, and colon and showed that only colonic administration increased GLP‐1 secretions in C57BL6 mice. Recently, Kamakura et al.^[^ ^102^ ^]^ demonstrated that 25–50 µM αLA significantly increased GLP‐1 secretions from STC‐1 and GLUTag cells. The same group also demonstrated that aLA at 100 mg kg^−1^ body weight was ineffective in reducing food intake, but higher doses of 200 and 500 mg kg^−1^ body weight significantly reduced food intake compared to control mice.^[^ ^91^ ^]^ In addition to fatty acids, their derivatives such as oleoylethanolamide and 2‐oleoyl glycerol also induced GLP‐1 secretion via GPR119 from GLUTag cells and humans.^[^ ^61^, ^62^ ^]^

### 3.4 Proteins and Amino Acids

Dietary proteins and their digestive products are one of the potent stimulants of GLP‐1 secretion as reported in animals’ and humans’ trials.^[^ ^103^ ^]^ A diet containing a higher amount of proteins increased satiety and reduced glycemia by inducing GLP‐1 and PYY secretion.^[^ ^104^, ^105^ ^]^ Dietary proteins induced GLP‐1 and PYY release which affect appetite‐regulating areas in the brain directly or indirectly via vagal afferents.^[^ ^106^ ^]^ Protein hydrolysates such as peptone, whey, and fish protein hydrolysates, have been reported to stimulate GLP‐1 release in both in vitro and in vivo studies.^[^ ^54^, ^107^, ^108^ ^]^

Several studies in animals and humans demonstrated that amino acids such as glutamine, tryptophan, and arginine are more effective in stimulating GLP‐1 secretion compared to other amino acids.^[^ ^109^, ^110^, ^111^ ^]^ GLP‐1 secretion induced by amino acids involves both basolateral and apical sensing mechanisms, indicating that amino acids‐induced GLP‐1 secretions follow absorptive and postabsorptive mechanisms.^[^ ^112^ ^]^ The enteric‐coated L‐glutamine (6 g) was ineffective in inducing satiety and glycemia, however higher dose of L‐glutamine (30 g) exerted beneficial metabolic effects in humans.^[^ ^31^ ^]^ It has been demonstrated that EC_50_ for glutamine‐triggered GLP‐1 secretion from primary L‐cells (∼0.2 m) is close to normal plasma glutamine concentrations (0.1–1 mM) in humans.^[^ ^113^ ^]^ Other amino acids such as glycine and alanine also stimulated GLP‐1 secretion from GLUTag cells through activation of the ionotropic glycine receptor.^[^ ^114^ ^]^

### 3.5 Other Substances

Capsaicin, a bioactive substance in chili peppers, stimulated GLP‐1 secretion from murine‐derived enteroendocrine cell line, STC‐1, through activation of transient receptor potential channels vanilloid subtype.^[^ ^52^, ^53^ ^]^ Another pungent product, allyl isothiocyanate from mustard has been reported to increase CCK secretion from STC‐1 cells.^[^ ^52^ ^]^ Polyphenols also stimulate GLP‐1 release. A tea component, epigallocatechin‐3 gallate, has been demonstrated to increase GLP‐1 release in an ex vivo study.^[^ ^115^ ^]^ Coffee polyphenol extracts induced GLP‐1 secretion from NCI‐H716 cells and elevated postprandial plasma active GLP‐1 levels in mice.^[^ ^116^ ^]^ Supplementation of resveratrol (5 weeks), a natural polyphenol produced by plants like red grapes and berries, elevated plasma active GLP‐1 levels after oral glucose tolerance test and increased intestinal GLP‐1 concentrations in mice fed with a high‐fat diet.^[^ ^117^ ^]^

In summary, stimulation of GLP‐1 via different nutrients is well established. However, these L‐cells are mainly located in the distal part of the human GI tract, which poses challenges for the nutrients to reach their site of action with regard to their therapeutic use. Therefore, effective systems for the delivery of nutritional compounds to the distal intestine are needed to stimulate endogenous GLP‐1 release in a sustained manner and inhibit appetite. Features and challenges of such a delivery system will be described in the following chapters.

## 4 Delivery Techniques Used for Nutrients

### 4.1 Requirements and Challenges in Colon‐Targeted Delivery of Nutrients

The oral route is the most common way to administer pharmaceutical compounds to the GI tract.^[^ ^118^ ^]^ Oral delivery has several advantages such as being noninvasive and easy to handle but suffering from low oral absorption due to acidic pH in the stomach, intestinal permeability, enzymes, microbiome mediated metabolism, and fluid volume.^[^ ^119^ ^]^ The epithelial lining of the intestine, pH of the GI fluids, and fluid volumes are the major barrier to the absorption of the bioactive compounds. Intestinal microvilli provide a large surface area for absorption but also an enzymatic barrier since their brush border is concentrated with digestive enzymes.^[^ ^120^ ^]^ Most of the bioactive agents in the nutrients are highly lipophilic and show poor bioavailability due to insolubility and low intestinal permeability in the GI tract.^[^ ^121^ ^]^ GI transit time plays an important role in the absorption, the small intestine transit time is quite steady between 4.3 and 4.6 h, whereas colonic transit time is greatly varied from 18 to 34 h in humans depending on the age.^[^ ^122^, ^123^ ^]^ The fluid volume decreases along the GI tract affecting the absorption of compounds, especially in the distal part of the intestine and colon. In healthy humans, it has been shown that food suppresses the mean fluid volumes in the small intestine compared to fasting conditions,^[^ ^124^ ^]^ and affects bile salts and digestive enzyme concentrations.^[^ ^125^ ^]^ The colon has two layers of mucus, the inner layer is anchored to the intestinal epithelial cells, and the outer layer is gel‐forming loose mucus, the habitat of the microbiome.^[^ ^126^ ^]^ The intestinal fluid volume affects the viscosity of the mucous layer, which is essential for defense mechanism against bacteria and influences cellular uptake of luminal contents.^[^ ^119^ ^]^

**Figure 3:**
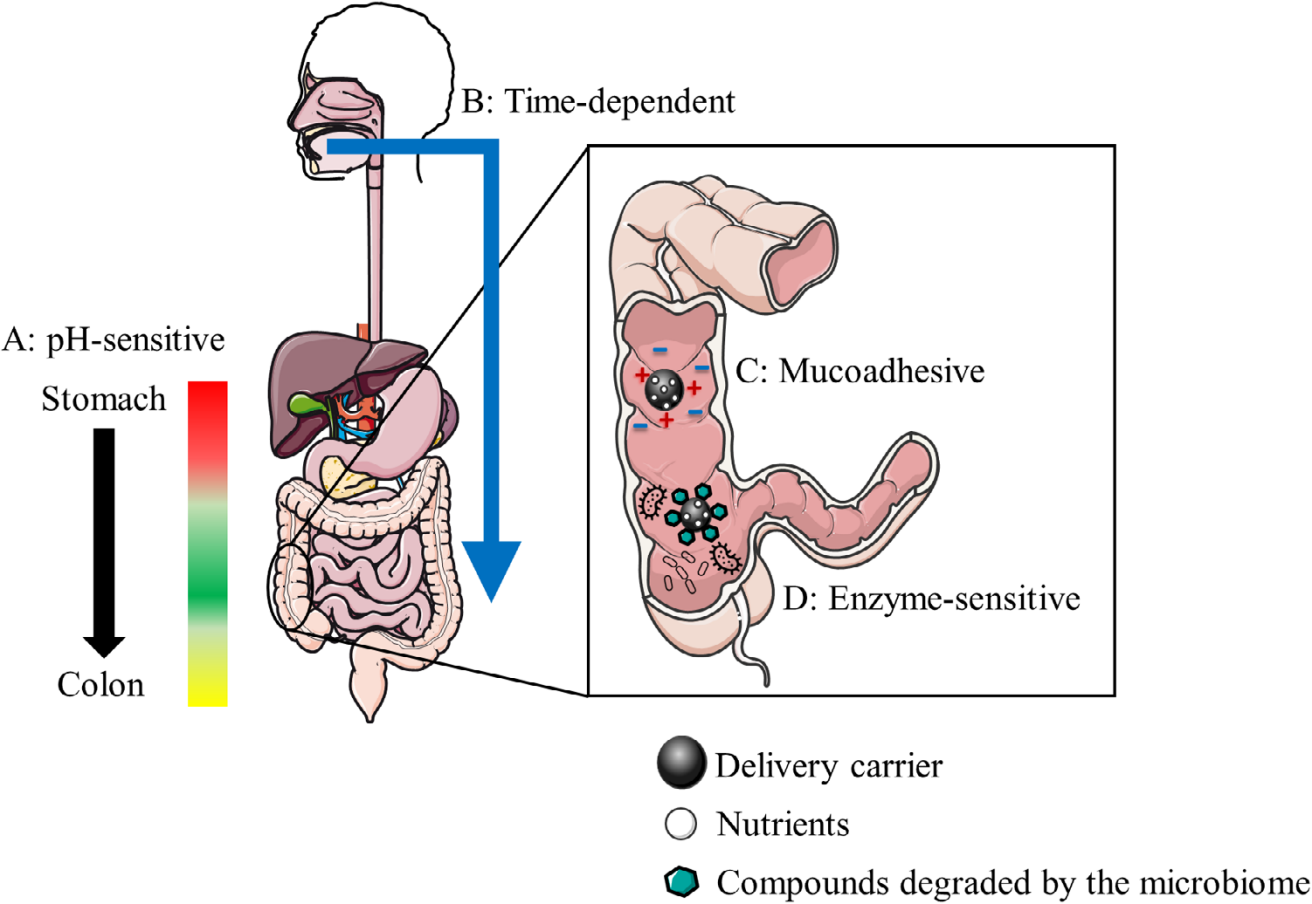
Examples of the colon‐targeted oral delivery of nutrients. A: The pH‐sensitive delivery system uses the existence of pH gradient in the GI tract. B: The time‐dependent delivery system utilizes transit time in the GI tract. C: The mucoadhesive delivery system uses the negatively charged colonic mucus surface. D: The enzyme‐sensitive delivery system utilizes enzymatic activity of colonic flora.

The incorporation of bioactive compounds into a carrier system is a way to overcome these barriers.^[^ ^127^ ^]^ The distal part of the human GI tract, with its low enzymatic activity and longer transit times, provides the best site for the absorption of bioactive compounds.^[^ ^128^ ^]^ Moreover, GLP‐1 and PYY secreting L‐cells are localized in the distal ileum and colon, thus administering nutrients to the distal GI tract will be more feasible for gut peptides release.^[^ ^9^ ^]^ The common strategies for colon‐targeted delivery systems are summarized in **Figure** **3**.

### 4.2 Materials Applied for the Colon‐Targeted Delivery of Nutrients

#### 4.2.1 pH‐Sensitive Delivery System

This approach utilizes the existence of a pH gradient in the different segments of the GI tract, which greatly varies in different species depending on feeding status (**Table** **1**). In mice and rats, the stomach pH slightly increases (pH 3–4) during fasting, however, in humans the pH decreases from 5 to 1–2.5 in the fasted state.^[^ ^129^, ^130^ ^]^ The pH of the proximal and distal small intestine in mice varies from 4.7 to 5.2 in the fasted state, while it is about 4.8 under fed conditions. Rats have slightly higher pH values in the proximal and distal small intestine than mice, i.e., 5.8–6.1 in the fasted state and 5.0–6.0 after feeding.^[^ ^130^ ^]^ In humans, the pH varies from 6.0 to 7.3 in the upper small intestine and 6.8–7.8 in the lower small intestine.^[^ ^131^, ^132^ ^]^ The colonic pH is generally lower than the small intestine. In mice, it is approximately 4.7–5.0 in the fasted state, and it decreases to 4.4–4.7 under fed conditions, while in rats it varies from 5.8 to 6.2 and 5.5–5.7 under fed or fasted state, respectively.^[^ ^130^ ^]^ The pH in the ascending colon of humans is about 5.2–6.7, and it increases up to 8.0 in the distal colon.^[^ ^118^, ^132^ ^]^ These varieties of pH values in the different GI segments can be utilized for achieving site‐specific delivery but vary according to the nutrition, and the minor pH difference between the small intestine and large intestine needs to be considered.

**Table 1:** The pH at the different parts of the gastrointestinal tract in mice, rats, and humans.^[^ ^113^, ^114^, ^115^ ^]^

Species	Mice		Rats		Humans
Fed/Fasted	Fed	Fasted	Fed	Fasted	Fasted
Stomach	∼3.0	∼4.0	2.2–4.0	3.0–5.0	1–2.5
Proximal small intestine	4.7–4.9	4.7–5.0	4.9–5.1	5.8–6.1	6.0–7.3
Distal small intestine	4.7–4.9	5.0–5.2	5.1–6.0	5.8–6.1	6.8–7.8
Proximal colon	4.6–4.7	4.9–5.0	5.4–5.6	6.1–6.3	5.2–6.7
Distal colon	4.4–4.5	4.7–4.8	5.7–5.8	5.8–5.9	5.2–8.0

In this approach, the delivery systems are coated with pH‐responsive enteric polymers, which have high solubility above pH 5–7 but are poorly soluble or insoluble in acidic pH.^[^ ^133^ ^]^ By combining the knowledge of the polymers and their solubility at different pH environments, delivery systems can be designed to carry payloads to the target site. Various pH‐sensitive polymers such cellulose acetate phthalate (CAP), hydroxypropyl methyl‐cellulose phthalate (HPMCP) 50 and 55, and copolymers of methacrylic acid and methyl methacrylate (e.g., Eudragit) are used for colonic targeting.^[^ ^134^ ^]^ Particularly, Eudragit are widely used in the colonic delivery due to mucoadhesive properties and pH‐dependent release; Eudragit E100 dissolve at pH under 5.0, while Eudragit L100–55 and Eudragit S100 soluble at pH over 5.5 and 7.0, respectively.^[^ ^135^ ^]^ In addition to the single‐layer polymer, the double‐layer pH‐dependent coating polymer (DuoCoat) was employed to achieve a shorter dissolution lag time and higher release rate at neutral pH.^[^ ^136^ ^]^ Hydrogels are biomaterials also used for colonic delivery for hydrophilic bioactive compounds due to high loading efficiency and simple preparation methodology.^[^ ^137^, ^138^ ^]^ The enzyme and pH‐sensitive hydrogels are most commonly used in colonic delivery due to substantial pH variations and the existence of colonic flora in the GI tract.^[^ ^139^ ^]^ Another interesting pH‐sensitive colonic delivery system is based on mesoporous silica particles coated with ε‐polylysine preventing the release of the cargo in the stomach.^[^ ^140^ ^]^ However, the pH‐dependent polymers have demonstrated significant variability in the release characteristics or even failure due to the different physiological factors (feeding status, fluid volume, GI motility, and disease status) between individuals.^[^ ^141^, ^142^ ^]^ To overcome the limitations of the pH‐dependent delivery system several strategies were introduced including a combination of pH‐dependent systems with either time‐dependent or enzyme‐triggered release systems.^[^ ^142^ ^]^

#### 4.2.2 Time‐Dependent Delivery System

Time‐dependent delivery system, also known as pulsatile drug delivery system, designed to release its contents after a predetermined time based on GI transit time. Delivery systems are designed with a lag‐time of no release for 5–6 h based on the assumption of relatively constant small intestine transit time of 3–4 h in the fasted state.^[^ ^119^ ^]^ For colon targeting, time‐dependent systems are often combined with a pH‐sensitive delivery system to prevent its degradation in the stomach and small intestine.^[^ ^143^ ^]^ The major challenge in designing a time‐dependent delivery system is the GI transit time, which is considerably different in subjects, depending on diet, age, and disease status.^[^ ^144^ ^]^ Organic polymers have been designed because of their tunable mechanical properties, biocompatibility, and biodegradability.^[^ ^145^ ^]^ Several photo‐crosslinked polymers like polyesters, polyanhydrides, polyethylene glycol (PEGs) polyurethanes, and their different copolymers have been utilized in a delivery system.^[^ ^146^, ^147^ ^]^ Hydrophilic polymers received considerable interest because they gradually swell over time and release its contents by erosion of the polymers.^[^ ^148^ ^]^ Polyester and polyanhydrides degrade hydrolytically, but polyanhydrides degradation is pH sensitive and stable in acidic conditions, which is beneficial for colonic delivery.^[^ ^149^ ^]^ Surface eroding polymers are favored for controlled release because their release characteristics depend on polymer degradation, on the other hand, in bulk erosion the release is via diffusion.^[^ ^150^ ^]^

#### 4.2.3 Mucoadhesive Delivery System

This approach targets the mucus layer of the GI tract, which is highly viscoelastic and adhesive consisting of mucin, lipids, and mucopolysaccharide.^[^ ^132^ ^]^ The mucous layer traps bacteria, viruses, and other toxic particles by electrostatic and/or hydrophobic interactions and serves as a protective surface.^[^ ^151^ ^]^ Sulfate and sialic acid in mucins make the colonic mucosa negatively charged.^[^ ^152^ ^]^ Mucosal adhesion is beneficial in colonic delivery since it promotes better contact with the mucosal surface for cellular uptake and release by extending the mean residence time in the colon.^[^ ^153^ ^]^ Cationic surface nanoparticles received considerable interest mainly due to the interaction between positively‐charged nanocarriers and negatively‐charged intestinal mucosa.^[^ ^152^ ^]^ Chitosan possesses mucoadhesive properties due to its positive surface charge that forms hydrogen and electrostatic bonds to the negatively charged sialic‐acid group of mucin.^[^ ^154^ ^]^ However, it has been demonstrated that anionic nanoparticles are better carriers due to lower electrostatic interaction with the mucus layer.^[^ ^155^ ^]^ Alginate and kappa‐carrageenan are anionic polysaccharides used for colonic delivery due to their mucoadhesive and antibacterial properties.^[^ ^156^ ^]^ A combination of these polysaccharides’ chitosan and alginate/carrageenan form polyelectrolyte complexes resistant to the acidic pH.^[^ ^157^ ^]^ Various hydrophilic polymers such as polyacrylic acid (Carbopol), carboxymethylcellulose (CMC), hydroxypropyl cellulose (HPC), and hydroxypropyl methylcellulose (HPMC) have excellent mucoadhesive properties due to non‐covalent and specific binding on the mucosal surface.^[^ ^158^ ^]^ Recently, it has been demonstrated that HPMC forms pores on the coating surface , which prevents degradation in the upper GI tract and sustained release of the compound in the colon compared to other mucoadhesive polymers including Carbopol and PEG.^[^ ^159^ ^]^

#### 4.2.4 Enzyme‐Sensitive Delivery System

This approach involves covalent linkage between the bioactive compounds and carrier system, which degrades mainly by the enzymatic activity of colonic flora.^[^ ^160^ ^]^ The major endogenous enzymes involved in the metabolism of ingested nutrients are listed in **Table** **2**. In the human GI tract, more than 500 bacterial species have been found, most of them are in the colon, while to a lesser density in the small intestine.^[^ ^161^ ^]^ The genetic and environmental factors such as diet contribute to the variation of microbiota composition in each individual.^[^ ^162^ ^]^ Furthermore, the gut microbiota contributes to enzymes that are not encoded by the human genome, and therefore have significant impacts on the metabolism of polysaccharides and polyphenols.^[^ ^163^, ^164^ ^]^ The enzymes and microbiome might affect the stability and releasing profile of carrier materials and will add to the variability of the response.

**Table 2:** The major enzymes involved in the metabolism of ingested nutrients

Enzyme	Produced by	Digest of
Amylase	Salivary glands, pancreas	Starch
Pepsin	Stomach	Proteins
Protease	Stomach, pancreas, small intestine	Proteins
Trypsin, chymotrypsin	Pancreas	Proteins
Lipase	Pancreas	Fats
Nuclease	Pancreas	DNA, RNA
Peptidases	Small intestine	Peptides
Maltase, sucrase, lactase	Small intestine	Saccharides
Nucleosidases, phosphatases	Small intestine	Nucleotides

Polysaccharides are decomposed by colonic flora including pectin, chitosan, inulin, and dextran, which have been used in the colonic delivery system.^[^ ^165^ ^]^ In addition, polymers, which degrade either in the presence of bacterial enzymes^[^ ^166^ ^]^ or under the low oxidation potential,^[^ ^167^ ^]^ are additional candidates for colonic delivery (e.g., azo polymer).^[^ ^128^ ^]^ When polymers reach the colon, the disulfide bridges broken due to the reducing environment of the colon,^[^ ^166^, ^168^ ^]^ thereby releasing the compound. For example, branch‐chained disulfide polymers based on the amino acid were synthesized with cysteines,^[^ ^169^ ^]^ minimizing side effects after degradation of the cysteine‐based polymers. The use of cross‐linked disulfide‐containing polymers may prove to be an effective approach to deliver compounds to the colon.

In summary, although several strategies have been applied and developed for colon‐targeted delivery systems, but further investigations are required to improve the colonic delivery of nutrients.

## 5 Future Perspectives

The prevalence of obesity and related diseases are alarming and the economic burden of health care is rising.^[^ ^170^ ^]^ Novel tools to prevent weight gain and excess energy intake are urgently needed. Utilizing nutrients to induce the endogenous gut peptides release to reach negative or neutral energy balance could offer a novel convenient way to prevent obesity and overweight.

Targeted drug delivery systems (TDDs) have been used for treating various chronic diseases namely cancer, diabetes, myocardial ischemia, atherosclerosis, asthma, Alzheimer's disease, and Parkinson's disease.^[^ ^171^ ^]^ Advanced TDDs have a vast impact on colon delivery because the delivery of protein and peptide‐related compounds are complicated owing to their polar nature and large size.^[^ ^172^ ^]^ Colon‐targeted drug delivery is a fast‐growing research interest to researchers to avoid the systemic absorption, toxicity, and increase drug concentration at the target site.^[^ ^172^ ^]^ It could become a promising way to develop natural compounds and pharmaceutical formulations to treat colon‐targeted diseases.

Especially nanocarrier‐mediated TDDs are the advanced strategy to deliver the natural compounds and nutrients due to their low bioavailability.^[^ ^173^ ^]^ Nanorobotic targeted drug delivery would be a great advancement in the coming decade due to self‐powered, digitally monitoring target delivery within the human body. This nanorobotics are acting as nanoinjectors of the target cell to overcome entering the target cell (endocytosed). It could be an advanced application in future engineering to deliver the drug to the target.^[^ ^174^ ^]^

Ileo‐colonic delivery of conjugated bile acids significantly improved glucose homeostasis and increased postprandial GLP‐1 in human subjects.^[^ ^99^ ^]^ Colon‐targeted delivery system has been proposed that can withstand enzymatic and chemical degradation in the upper GI tract and release their payloads in the colon. Several studies reported that targeted delivery reduces systemic exposure and required lower dose of the compound for therapeutic effects compared to non‐targeted administration.^[^ ^175^, ^176^ ^]^

TDDs could be utilized to assist the nutrients to the distal GI tract and improve their effects on the endogenous secretion of gastrointestinal peptides. Applying TDDs would induce sustained endogenous peptide secretion, reduce the needed daily dose of compounds, and cost. The nutrients may be formulated with the delivery systems so that they could be mixed with either liquid or some type of snack, e.g., yogurt, and taken in between or prior to the meals. In addition, the delivery systems would mask their payload taste and unpleasant smell, increasing the usability in appetite management.

## Conflict of Interest

The authors declare no conflict of interest.
